# Optimality of Human Contour Integration

**DOI:** 10.1371/journal.pcbi.1002520

**Published:** 2012-05-24

**Authors:** Udo A. Ernst, Sunita Mandon, Nadja Schinkel–Bielefeld, Simon D. Neitzel, Andreas K. Kreiter, Klaus R. Pawelzik

**Affiliations:** 1 Department of Neurophysics, Institute for Theoretical Physics, University of Bremen, Bremen, Germany; 2 Department of Theoretical Neurobiology, Institute for Brain Research, University of Bremen, Bremen, Germany; Indiana University, United States of America

## Abstract

For processing and segmenting visual scenes, the brain is required to combine a multitude of features and sensory channels. It is neither known if these complex tasks involve optimal integration of information, nor according to which objectives computations might be performed. Here, we investigate if optimal inference can explain contour integration in human subjects. We performed experiments where observers detected contours of curvilinearly aligned edge configurations embedded into randomly oriented distractors. The key feature of our framework is to use a generative process for creating the contours, for which it is possible to derive a class of ideal detection models. This allowed us to compare human detection for contours with different statistical properties to the corresponding ideal detection models for the same stimuli. We then subjected the detection models to realistic constraints and required them to reproduce human decisions for every stimulus as well as possible. By independently varying the four model parameters, we identify a single detection model which quantitatively captures all correlations of human decision behaviour for more than 2000 stimuli from 42 contour ensembles with greatly varying statistical properties. This model reveals specific interactions between edges closely matching independent findings from physiology and psychophysics. These interactions imply a statistics of contours for which edge stimuli are indeed optimally integrated by the visual system, with the objective of inferring the presence of contours in cluttered scenes. The recurrent algorithm of our model makes testable predictions about the temporal dynamics of neuronal populations engaged in contour integration, and it suggests a strong directionality of the underlying functional anatomy.

## Author Summary

Since Helmholtz put forward his concept that the brain performs inference on its sensory input for building an internal representation of the outside world, it is a puzzle for neuroscientific research whether visual perception can indeed be understood from first principles. An important part of vision is the integration of colinearly aligned edge elements into contours, which is required for the detection of object boundaries. We show that this visual function can fully be explained in a probabilistic model with a well–defined statistical objective. For this purpose, we developed a novel method to adapt models to correlations in human behaviour, and applied this technique to tightly link psychophysical experiments and numerical simulations of contour integration. The results not only demonstrate that complex neuronal computations can be elegantly described in terms of constrained probabilistic inference, but also reveal yet unknown neural mechanisms underlying early visual information processing.

## Introduction

The human's analysis and perception of complex natural scenes under greatly varying environmental conditions is robust and rapid. This remarkable ability of our brain relies on various interacting processes which can be assumed to build representations of visual objects from the information contained in localized image patches. A very elementary process in this context is contour integration, where sets of colinearly aligned line segments or edge elements are merged into coherent percepts of contours.

Contour integration is useful for identifying boundaries of potential objects in a visual scene, and therefore important for performing image segmentation and object recognition. Humans and primates are remarkably efficient in integrating contours even if the edges of a contour are not perfectly aligned or if parts of the contour are occluded by other image components. Thus uncovering theoretical principles and neural mechanisms underlying contour integration is an important step towards understanding visual information processing in the brain [1]–[3].

Psychophysical studies have investigated the impact of various stimulus parameters on contour integration. For example, they quantified how contour integration performance depends on contour curvature [4], on the distance between consecutive contour elements [5], [6], on the deviation from a perfect alignment of the oriented elements to the contour path [4], or on the spatial frequency of the elements [7].

The first attempt to put such observations into a coherent framework was made by a group of psychologists [8], [9]. They formulated the Gestalt laws for describing the principles according to which the visual system groups local image features into coherent percepts. The corresponding principle for contour integration is termed the ‘law of good continuation’, stating that line segments which are aligned colinearily or curvilinearly are bound together. This idea was later formalized by introducing the ‘association field’ (AF) [4], which specifies how strongly the visual system associates two line segments with a particular configuration of positions and orientations as belonging to one contour.

Ideally, a theory of contour integration should predict perceptual behaviour for arbitrary configurations of oriented image patches. Here, we explore if an approach based on ‘generative models’ can quantitatively predict human contour detection. Generative models derive from the classical perspective that considers perception as inference [10]. They are statistical models specifying how a stimulus might be generated from the presence or absence of particular elementary causes or objects in a scene. Knowing the generative process enables an observer (i.e., the brain) to perform inference on such a stimulus.

In the context of visual perception, this perspective has recently shown to be useful for understanding and modeling multisensory cue integration [11], [12]. In these investigations the objective of perception was specified by a particular task which essentially requires computations on only two sensory cues. In comparison, contour integration is far more sophisticated, performed on many sensory variables in parallel, and according to an objective which is yet not known in a quantitative, mathematical sense. A promising conceptual idea for closing this gap is given by the observation that the association field can be reinterpreted as a conditional link probability between two oriented line segments [13], [14]. This interpretation can be used to define a contour generation process that relies on similar conditional probabilities. Formulated as a generative model for contours, it yields a specific statistics of stimuli comprising oriented line segments. By inversion of this generative process, contour integration is now reduced to an optimal inference problem, namely the computation of the probabilities for an element to belong to a contour. A thorough formalization of this idea was performed by Williams and Thornber [13] who used it to explain certain visual illusions.

**Figure 1:**
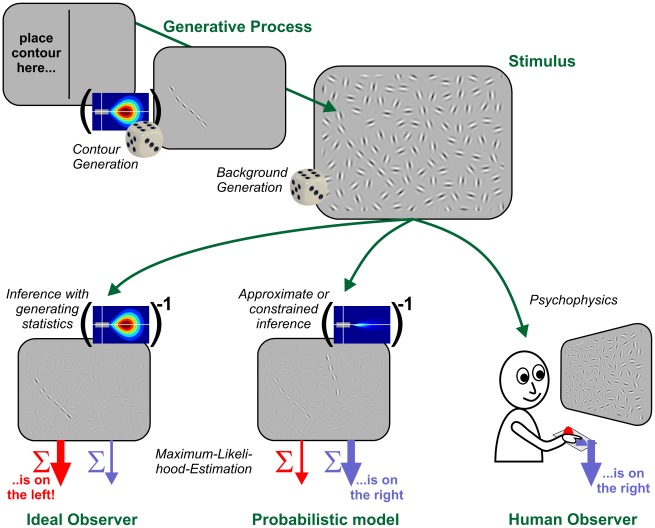
Framework for combining theory, modeling and psychophysics to study contour integration. Upper row, contour creation: A contour is created either on the left or right hemifield of a computer screen by a Markov random process using a suitably defined association field (AF, in brackets) for specifying the transition probabilities. Adding randomly oriented, similarly spaced background elements effectively hides the contour and completes a stimulus. Lower left column, contour integration: The ideal algorithm for contour integration uses knowledge about the generating process (i.e. the same AF as used in generating the contours, in brackets), to perform inference on a stimulus. For each edge, it computes the probability of being the first (or last) element of a contour created by the generating Markov process. The likely position of a contour is finally determined by maximum-likelihood estimation on the sum of these probabilities for each hemifield. Lower middle and right column, comparison to humans and probabilistic models: In our paradigm, the ideal contour observer serves as a benchmark for human contour detection, which is probed using the same stimuli under time constraints. At the same time, the inference algorithm of the ideal observer suggests a class of probabilistic contour integration models in which we search for the optimal model which best explains human behavior and performance. Note that ‘optimal’ does not mean that the contour integration model strives for an optimal contour detection performance: it should also make the same errors as human observers, as in this illustrative example, where a shorter ‘chance’ contour in the background is judged more salient by the human subject.

The present work pursues an integrative approach linking theory, modeling and psychophysical experiments. It aims at explaining human contour integration and decision behaviour as optimal inference in a mathematically exact and quantitative manner (see **Fig. 1**). By extending the theoretical framework of Williams and Thornber [13], we define a class of generative models for contour integration from which we construct mathematically well–defined ensembles of test stimuli for psychophysical contour detection experiments. Using behavioral data collected from five human observers, we subsequently identify the parameters of the generative model which most closely explains human decisions for each stimulus. We find that these parameters match the findings from previous empirical work. An extensive statistical analysis reveals that the best–matching model reproduces practically all systematic behavior among our subjects. From the particular structure and dynamics of our model, we derive predictions about putative neural mechanisms realizing probabilistic contour integration in the brain. Finally, we discuss these findings in comparison with physiological and anatomical evidence from visual cortex.

## Results

### A generative model of contour creation and integration

The statistics of contours in natural images is highly complex, and there is no complete description that could be taken as a starting point for a modelling study. The best information available is from studies with human observers who were instructed to redraw contours in a set of natural images [15]–[18]. However, this statistics was only extracted for pairwise edge configurations, and is only available as a tabulation and not in a closed–form expression. We instead chose to employ the probabilistic framework by Williams and Thornber [13] and defined contours as being generated by a Markov random process (details in Methods section): To create a contour of length 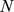, one places its first edge 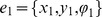 with random angle 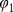 at a random position (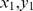). The second edge 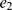 of the contour is then placed by randomly drawing its position and angle from a conditional probability density 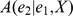 with (as yet unspecified) parameters 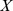. This process is iterated until the final, 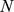-th edge has been placed. Note that we actually define the parameter 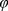 as a direction extending over the full circle 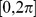, rather than representing an orientation only. This definition is necessary for constraining contour creation to proceed along a chosen, general direction. It prevents the creation process from turning around by 180 degrees when placing successive edge elements. For a more elaborate justification and discussion of this property, we refer to [13]. Note that we would also like to understand the term ‘edge’ in a more general sense as any realization of an image patch, which is *localized* at a position 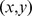 and has an *orientation* 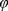. This definition encompasses line segments and luminance borders, as well as the Gabor patches which we used for rendering the stimulus configurations generated by our probabilistic model.

**Figure 2:**
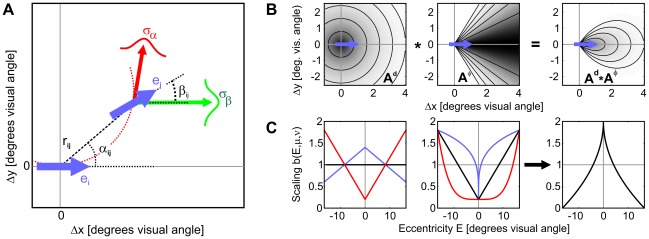
Parameters and geometry of association field, and eccentricity scaling. (A) Geometrical relation between edges 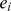 and 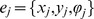 and their relative coordinates 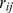, 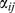 and 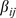. The red arrow indicates the direction edge 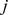 should have for a perfect co-circular continuation of a contour through 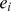 and 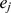. Conditional link probability 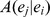 (the ‘association field’) depends on the deviation of 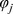 from this direction with a scale of 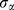 (in red). In addition, link probability also depends on the difference 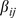 between the directions of 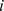 and 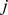 on a length scale 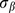 (in green). (B) The association field 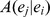 is defined as a product of a radial part 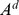 and an angular part 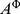 (see Methods). The starting edge 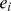 with direction 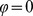 is shown as the blue arrow in the center of the coordinate systems. Left, the radial part 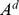 is shown in dependence on the distances 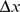, 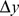 to the destination edge. Center, the angular part averaged over all destination directions 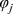. Right, the product of the distributions in the left and center graphs. Grey scale is proportional to link probability, normalized to 1 (darker shades indicate higher values). Parameters of all sketches are taken from optimal model which was fit to explain the psychophysical data. (C) Edge salience 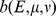 in dependence on edge eccentricity 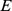 and parameters 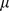 and 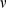. Left, for a constant 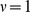 the parameter 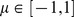 controls the slope (black, 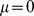; blue, 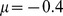; red, 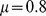). Center, for a constant 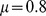 the parameter 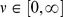 controls the concavity (black, 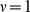; blue, 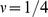; red, 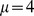). Right, the scaling 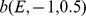 obtained by fitting the probabilistic contour integration model to human behavior.

**Figure 3:**
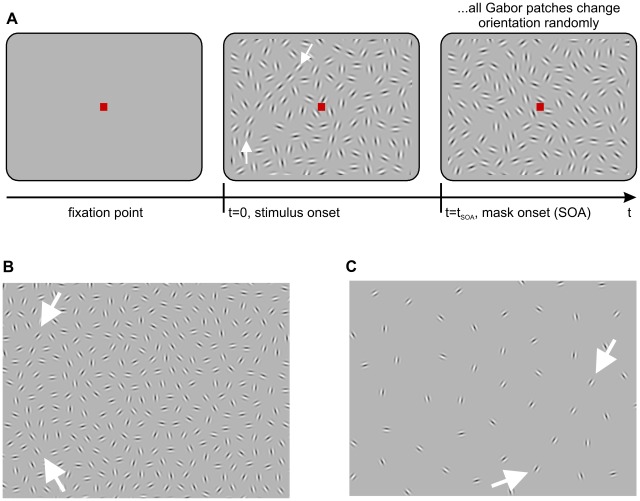
Contour detection paradigm and stimulus parameters. (A) Each trial started with the appearance of a fixation point. Subsequently, a stimulus was presented with a contour hidden in the left or right hemifield of the screen. This stimulus was masked after a time 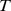 after stimulus onset (SOA). The mask consisted of edge elements at the same positions, but with random orientations. Edge elements were rendered as Gabor patches with random phases. For better visibility, the size of the Gabors was scaled by a factor of two in this illustration. (B) Sample section of a different stimulus with a straight, but jittered contour of 10 elments, smallest mean edge distance. (C) Sample section of a stimulus with the largest used edge distance and a contour of 4 elements. In all panels, the location of the contour is indicated by white arrows, which were absent in the real experiment.

Reasonable choices of 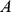 promoting features like colinearity and cocircularity [19] (**Fig. 2** A,B), yield contour samples which look quite ‘natural’ (**Fig. 3**). In particular, these samples are perceived by humans as contours and are salient when hidden among distracting elements. The probability density 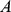 may be identified with the AF [4] which is commonly used in psychophysical literature to quantify how strongly a given configuration of two edges provides evidence for the presence of a contour. Given the distribution of contour elements and their total number are known a priori, the properties of 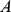, parametrized by 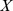, fully define the contour statistics.

This probabilistic framework for contour generation not only provides contours with a well-defined statistics, but also implies an ideal model for contour detection: Suppose that the 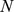 contour edges are hidden in a field of 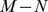 randomly oriented distractor edges. Given the generating AF 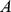 was known, one can now compute the likelihoods 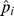 that edge 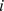 is the starting edge of a contour with length 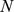. This is done by first constructing a matrix 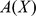 of the pairwise association probabilities for all edges combinations (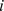, 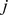) by sampling from 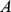 via 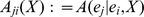 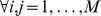. The likelihoods 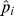 are then given by an ordinary matrix multiplication (with 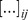 denoting the matrix element from the 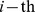 row and 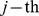 column from the expression inside the square brackets),
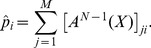
(1)Here we adapted the basic framework from [13] to contours of finite length which consist of a discrete set of elements. With few modifications it is equally possible to handle continuous contours, or to integrate closed contours [13]. Note, that this algorithm computes the true likelihoods only if the assumptions about the underlying process are correct. In other words, it only then realizes an ideal observer when contour integration is performed with the same parameters 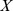 that were used for contour generation. When applied with deviating assumptions it may still be used to perform approximate inference, which, however, would be prone to systematic misestimations.

The position of the contour can be estimated from the location (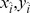) of the edge 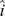 with the largest likelihood of having been the starting edge of the contour (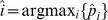, maximum likelihood estimator). By eqn. (1), all possible contour paths are exploited in parallel. It is related to iterative Bayesian estimation in the sense that each matrix multiplication iterates a prior that contains the starting edge likelihoods for contours with 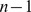 elements into a posterior that contains the likelihoods for 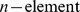 contours. In total, 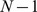 iterations are required when looking for contours of 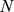 edges.

In the context of two–alternative forced choice (2-AFC) experiments it is less interesting to determine the precise position of a contour than to infer which one of two different stimuli 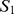 and 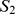 is more likely to contain a contour. Given the corresponding matrices 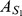 and 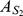 for stimulus configurations 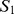 and 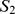, respectively, one first computes
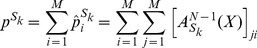
(2)and then compares which of the likelihoods 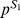 or 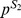 is larger.

In addition, we introduce a scaling factor 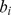 for each edge 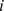. This factor is used to cover situations in which edge elements have different degrees of visibility. 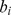 would be low if, for example, an edge is fuzzy, has a low contrast, or posseses no certain orientation, like at the border of a cloud in a natural scene. In a probabilistic framework 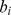 can be interpreted as a likelihood for the presence of an edge 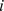 [13], which modifies the original contour integration algorithm eqn. (1),
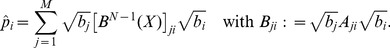
(3)Also 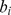 is part of the generative model and thus might depend on its parametrization 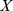, 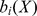.

In our paradigm, the objective for contour integration is to infer the likelihoods for (starting) edges being part of a contour, taking the observation of their orientations and positions as available evidence. By exploiting all knowledge about the statistical nature of contours contained in the AF, eqn. (3) realizes an ideal contour observer which performs iterative Bayesian estimation on the evidence provided by the edges in a stimulus. This ideal observer not only serves us as a benchmark for humans and models performing the given task: by assuming that human contour integration follows a similar objective, eqn. (3) describes a suitable probabilistic model class which we can require, by means of fitting their parameters 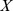, to reproduce human behavior as well as possible.

### Psychophysical contour detection experiments

We performed psychophysical experiments using the stimuli generated by our models. While our paradigm is similar to previous studies, our approach is conceptually different: we used a precise mathematical definition of edge configurations for generating contour stimuli, providing us with ideal observer models for integrating these contours. These models then served us as a benchmark for both, average human performance and individual human decision behaviour.

In a 2–AFC paradigm human subjects had to detect a contour which had been placed either into the left or into the right hemifield of a computer screen (**Fig. 3** A). The contour was hidden among randomly oriented distractors, which had been placed such that the only information left about the location of the contour was in the relative alignment of the contour's edges. Since we do not know a priori which exact parameters 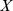 for our association field are best suited to match human contour integration, we choose different combinations 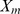 to systematically probe human behavior and to vary the difficulty of the task. In particular, we varied alignment of edges and curvature of the contours (**Fig. 3** B) by changing the length scales of the AF 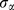 and 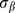, respectively (see Methods). In addition we varied mean inter-edge distance from 1.2 to 3.6 degrees of visual angle while holding the spatial extension of the contour constant, resulting in contours from 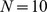 down to 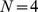 edges, respectively (**Fig. 3** C). For studying the temporal dynamics of contour integration, all stimuli were shown for varying time periods of 20, 30, 60, 100 and 200 ms. After this period (stimulus-onset asynchrony, SOA), masks were presented which consisted of edge elements located at the same positions, but with randomly assigned orientations. Stimuli from AFs with different underlying parameter sets 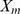 were presented in random order, which varied for each subject (for details of all procedures, see Methods section).

**Figure 4:**
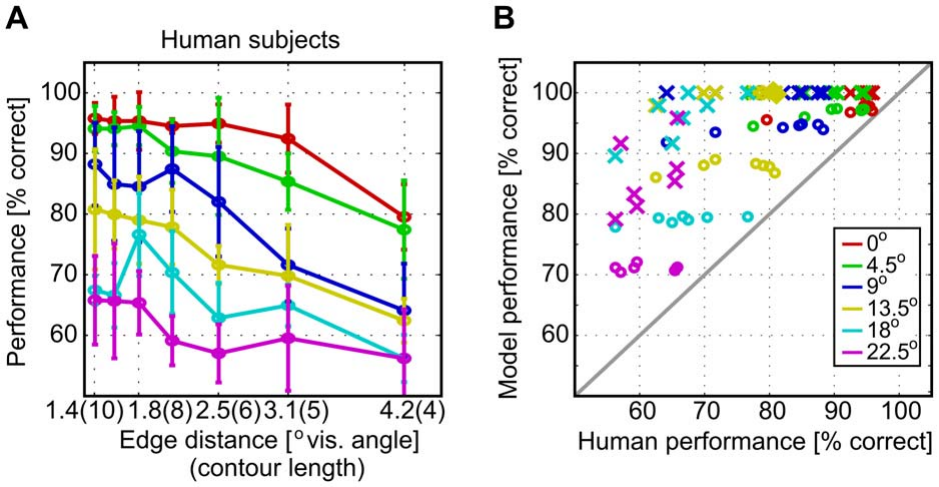
Contour detection performances. Comparison of contour detection performance in percent correct (A) for human observers to (B) the ideal and the optimal models. The performances are shown in dependence on inter–element distance (i.e., total number of edges in a contour) and on the alignment parameters 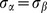 of the AF (color legend as inset to (B)). The psychophysical data for an SOA of 200 ms in (A) was averaged over 5 human observers, with the vertical bars denoting standard errors. In (B), model performances (ideal model: crosses, optimal model: open circles) for all contour ensembles (i.e., all jitters 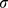 and contour lengths 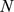) are plotted against the corresponding human performances. In this scatter plot, all points above the solid line indicate model performance being above human performance. Detection performance for the optimal model was averaged over 5000 samples from each contour ensemble, instead of using only 48 samples as in the actual experiment.

**Figure 5:**
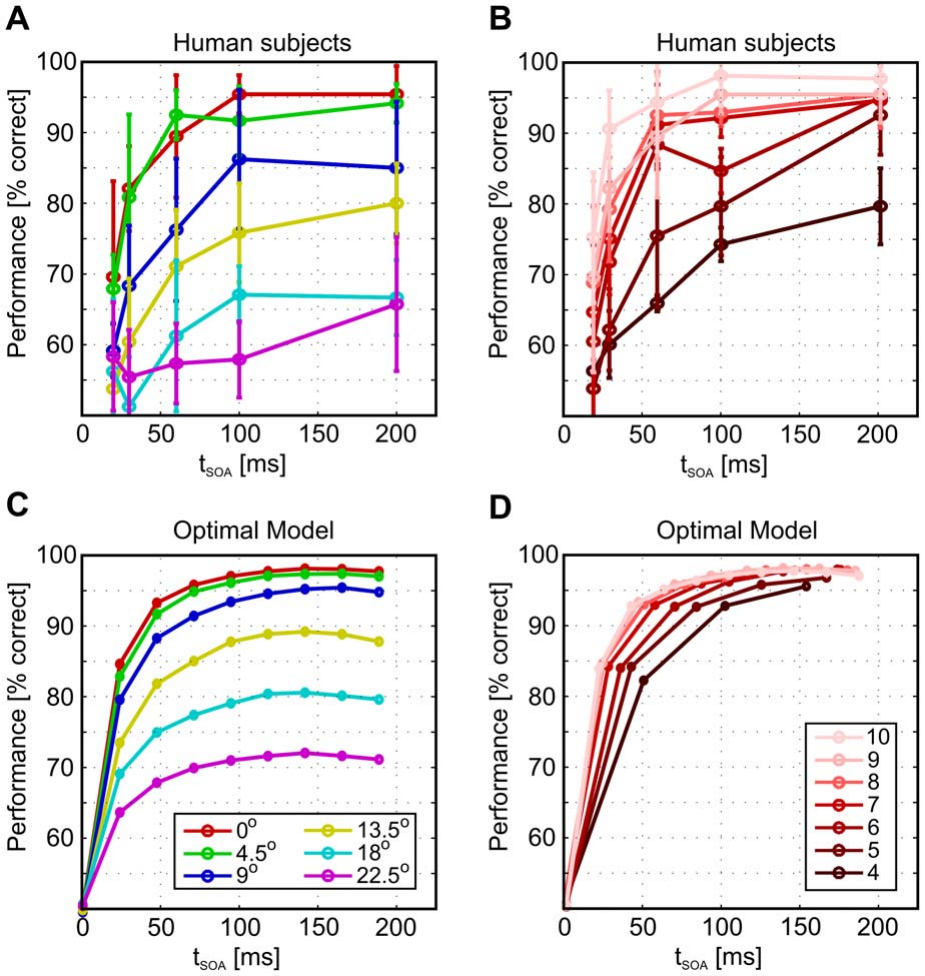
Temporal aspects of contour detection. Psychophysical contour detection performances in dependence on SOA in the upper row are compared to performance of the optimal model, which best matches human behaviour, in the lower row. Iterations performed in the optimal model were rescaled to time by assuming a constant propagation speed mediated by the AF interactions (corresponding to 13.9 DVA per 200 ms, which was the average length of all contours in the stimulus ensembles). (A) and (C) show performances for different AF alignment jitters, for contours of length 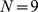 (color legend as inset to (C)). (B) and (D) show performances for different inter–element distances which are inversely proportional to the total number of edges in a contour, for an AF jitter of 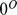 (color legend as inset to (D)). Detection performance for the optimal model was averaged over 5000 samples from each contour ensemble, instead of using only 48 samples as in the experiment, to yield a better statistics and smoother curves.

**Fig. 4** A and **Fig. 5** A,B summarize our experimental findings. We first focus on the data for an SOA of 200 ms (**Fig. 4** A): As expected, and in accordance with previous investigations [4], contours were more difficult to detect if edge alignment is subject to jitter and if contour curvature increases. Performance also decreased for increasing edge distances, but less strongly for straight contours. For such contours, almost perfect contour integration was still possible with inter-element distances up to 2.7 degrees of visual angle. **Fig. 5** A,B shows how contour detection performance improves with an increasing SOA between target and mask. Again, lower performance is observed for higher jitters or larger element distances, but the general shape of the curves is very similar. Surprisingly, we found considerable detection performances also when SOAs were as low as 20 ms. These results suggest that contour integration is a very fast process requiring long-ranging interactions between orientation detectors in visual cortex.

For comparison, we performed contour integration with the ideal observer model eqn. (3) on the same stimuli as used in the experiment. As we had perfect knowledge about orientation and position of each edge in the stimuli, the factors 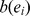 were all set to 1. By construction, the performance of an ideal observer must be superior or equal to any other observer, including human subjects. **Fig. 4** B (crosses) clearly shows that this is indeed the case, and that the ideal observer performs much better than humans. This large difference might be explained by a mixture of the following four factors:

1. Human observers might be subject to (decision) noise which is external to the contour integration process, whereas the ideal observer is noise–free.
2. Information available to the ideal observer and to human subjects could be different.
3. Objectives of the human observers could be different, e.g. our chosen definition of contours could substantially diverge from which edge configurations humans interpret as contours.
4. It might be impossible for the brain to actually perform the computations needed for (approximate) inference in the given task, e.g. because of neurophysiological and anatomical constraints.

To which extents do these factors explain the observed differences in performance?

Using a detailed statistical analysis of human and ideal observer decisions (introduced in the next subsection), we will show that the factor noise contributes only to a small extent. In contrast, the factor available information is much more important. Information provided to the model and to human observers is clearly different, because the ideal model eqn. (3) uses for each stimulus the exact AF parameters 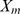 that were used for creating the hidden contour, while subjects in our experiment did not have this information. They did not know which kind of contour to expect in the next stimulus, because it was selected from the stimulus pool in a random order (see Methods). In consequence, the question arises whether one can find a *different* inference model from the *same* class of optimal models, however, with a fixed *single* set of parameters 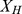, that can fully explain human behavior for all stimuli used in our experiment. Information available to this constrained model and to humans would then be identical. When applied to stimuli from an ensemble generated by an AF with different parameters, such a model would clearly no longer be ideal, but note that it would still represent an optimal estimator when used for contour stimuli from the ensemble that corresponds to its AF. If successful, this search would thereby yield an exact mathematical quantification of the assumed probabilistic objective for contour integration in human observers. Namely, it would reveal both, optimality per se and the specific ensemble of stimuli for which optimality holds, and it also would exclude computational constraints as reason for the observed difference in ideal and human performance.

To constrain the search for this ‘optimal’ model as strongly as possible, we will now introduce a novel statistical measure which quantifies how well a model predicts systematic human behavior. This measure extends beyond the usual approach of comparing average detection performance.

### Quantifying human decisions

The usual criterion for evaluating contour integration models is performance in correctly detecting contours (e.g. [16]). We have to require that a model at least reaches human performance. It may even exceed human performance substantially, as human decisions are usually subject to a fair amount of noise. Hence for assessing how well a model 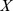 explains human performance, we determine the fraction 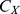 of stimulus sets 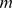 in which the model reaches or surpasses mean human performance. A stimulus set 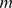 hereby refers to the parameters 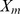 of the generating process.

A less obvious, but much stronger criterion is to consider any unexpected or excess correlations within the decisions of different human subjects to one specific stimulus set. Here we aim at a measure that reflects the decisions that are common to different subjects. This measure would more sensitively quantify the constructive contributions of contour integration to behaviour as compared to the simple performance measure. Performance is strongly influenced by the general difficulty of a task, and by destructive sources of noise which could be external to the processes underlying contour detection.

To illustrate our approach consider a particularly simple example: Suppose that two human observers try to detect contours in a set of 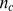 stimuli with the same statistical properties. Let us further assume that both observers reached the same performance 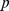 in detecting a contour correctly. If detection errors are made randomly and independently of a particular stimulus, for example through noise in the contour integration or decision process, we expect to find on average 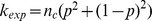 identical responses. We can now compare 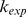 to the actually measured number of identical responses 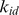. If on average over different stimulus sets, 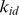 turns out to be significantly larger than 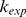, we can conclude that the two observers are more strongly correlated than expected under the independency assumption.

With this heuristics in mind, we can now more generally derive our measure of excess correlations: we first compute the expected distribution 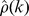 over the number of identical decisions 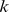 in one stimulus set, assuming independent detection errors (see Methods section). Using the actually measured 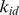, we then calculate the probability that sampling from 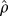 would yield a lower or equal value for 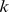. Finally we average these probabilities for different stimulus conditions, thus obtaining a measure 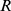 for excess correlations. This measure yields 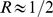 if the distribution of 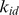
's is similar to 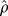. Any value of 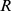 being significantly larger than 1/2 confirms the existence of decision correlations not explained by mean performances. The symbol 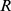 was choosen because our measure is related to the integral over a ROC curve, quantifying the distance between an expected distribution with an actually measured distribution.

Applying this analysis to pairs of human subjects, we found the values shown in **Table 1**. Apart from subject 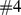, excess correlations are similar between pairs of subjects. Averaging over observer pairs yields a value of 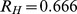, which is significantly larger than 0.5 (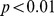, 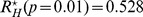). Significance was assessed by performing the same analysis on surrogate data generated by shuffling human decisions over the 48 stimuli for each stimulus ensemble. This procedure kept mean performance for each observer and stimulus ensemble constant, and allowed us to compute a value 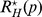 which correlations had to exceed to be considered statistically significant w.r.t. the corresponding 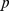-value. This result shows that human responses are far more correlated than expected from their average performances. It implies that particular stimuli, or whole stimulus subsets in a contour ensemble are either much easier, or more difficult to detect than others. For finding a good model of human contour integration, this means that additional information in correct or erroneous decisions can be exploited which is not contained in observer performance.

**Table 1:** Excess correlations 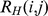 between all subject pairs 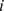, 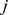.

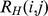	Subject 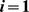	Subject 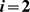	Subject 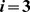	Subject 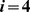	Subject 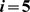
Subject 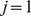	–	0.686	0.694	0.616	0.689
Subject 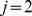	–	–	0.690	0.650	0.629
Subject 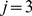	–	–	–	0.623	0.723
Subject 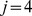	–	–	–	–	0.658
Subject 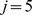	–	–	–	–	–

Excess correlations 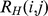 between all subject pairs 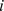, 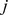. Note that by definition, 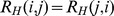. All values are significantly different from 0.5 (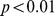).

If the ideal observer was a good model whose superior performance is solely a consequence of being not subject to noise, it would fully capture this systematic behaviour, and (besides its far higher performance) reveal equal or higher excess correlations when its decisions are compared with human decisions. However, evaluation of 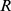 between human and ideal observer yields a value of 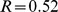 only, which is very close to chance level and far from 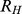.

In essence, we need a different model which may have lower performance in our specific task, but must have higher predictive power for human behaviour. Using a model that is not ideal in the sense that it deviates from the process that generated the contours will lead to systematic misdetections in a 2–AFC setting, which is one possible cause of the human's excess correlations. In the following, we will use mean performances 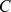 and excess correlations 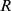 to systematically search for such contour integration models with *fixed* parameters which quantitatively and individually explain human behavior in *all* experimental conditions.

### A generative model for human contour integration

The excess correlations of 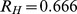 among human decisions constitute a benchmark for any proposed model 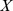: Instead of comparing pairs of two human observers, we will now compare a model 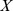 to human observers and require the excess correlations 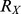 to reach the same value as 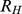. As mentioned above, in contrast to human observers a model is not subject to external noise affecting the decisions. This makes a direct comparison of excess correlation values 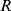 problematic because it will necessarily lead to higher values in 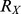. This statistical bias can be reduced by constructing a prototypical, noise-free human observer 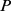. Its excess correlations 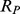 with the real, ‘noisy’ human observers (details see Methods) constitute a more stringent benchmark value for the noise-free models. For our data, we obtained 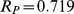 (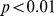, 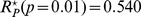).

We already explained that one reason for human observers exhibiting 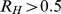 could be that a stimulus ensemble obtained from one generative process contains subsets of contours which are consistently easier to detect than other subsets. This is indeed the case in our experiment, where each contour ensemble contains contours placed at different eccentricities from the fixation spot. It is known that for humans, contours close to the fovea are more easy to integrate [20]–[22] than in the periphery, whereas the ideal model is translationally invariant and thus unaffected by the placement of a contour.

By searching for a model 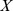 with 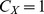 and 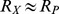, we favored models that replicate generic human behavior including correct but also erroneous decisions, rather than looking for an algorithm which has only the same, or higher, average contour integration performance. During our search we remained within the same class of (optimal) probabilistic models, but incorporated plausible constraints that relate to available prior knowledge. If successful, such a strategy will ultimately allow to explicitly state a probabilistic objective for human contour integration.

For finding an optimal model, we focused on two major determinants shaping human contour integration which have been identified by previous work [4], [23], [24]:

- Shape of the association field (AF): Although stimuli in our experiment were drawn from different AFs, we hypothesize that a single, general-purpose AF will be sufficient to model human contour integration. For parametrisation, we chose the product of von-Mises functions eqn. (9) which we originally used to create the stimuli (see methods section), but varied the alignment and curvature parameters 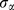 and 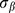 independently (**Fig. 2** A,B). For the radial part 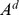 of the AF (**Fig. 2** B, left), we used an exponentially decaying function with spatial constant 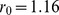 degrees of visual angle,
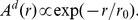
(4)
- Modulation of edge saliency with eccentricity: Contour integration performance strongly depends on mean contour eccentricity [20]–[22]. In our data, error rate on average increased from about 27 to 44 percent when eccentricity increased from about 2 to 11 degrees of visual angle (SOA = 200 ms).

The source for this effect may be rooted in the cortical magnification factor [25], which decreases with eccentricity. This leaves less neurons per unit area of the visual field providing information about a stimulus, leading to more noisy representations of visual features. In a task with a short SOA, detectability of edges would hence decrease with eccentricity. In our framework this is modeled by decreasing the scaling factors 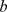 (see eqn. (3) for contour integration. With eccentricity 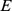 defined as the Euclidean distance from the fixation spot, we parametrized the dependency of 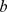 on 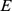 by
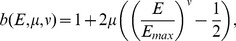
(5)using the two parameters 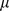 and 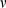 for systematically varying this function (**Fig. 2** C). The amplitude 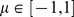 determines how strongly 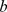 varies with eccentricity (**Fig. 2** C, left), and the exponent 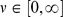 regulates how steeply 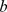 changes with eccentricity (**Fig. 2** C, center). For 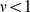, 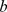 is concave down, and for 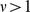, 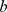 is concave up. 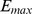 denotes maximum eccentricity in our setup which was 16.66 degrees of visual angle. For the special choice 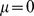 and 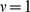, 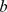 is constant and eqn. (3) would be identical to eqn. (1).

The four parameters 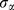, 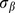, 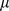, and 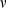 now uniquely determine the model candidates 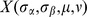.

**Figure 6:**
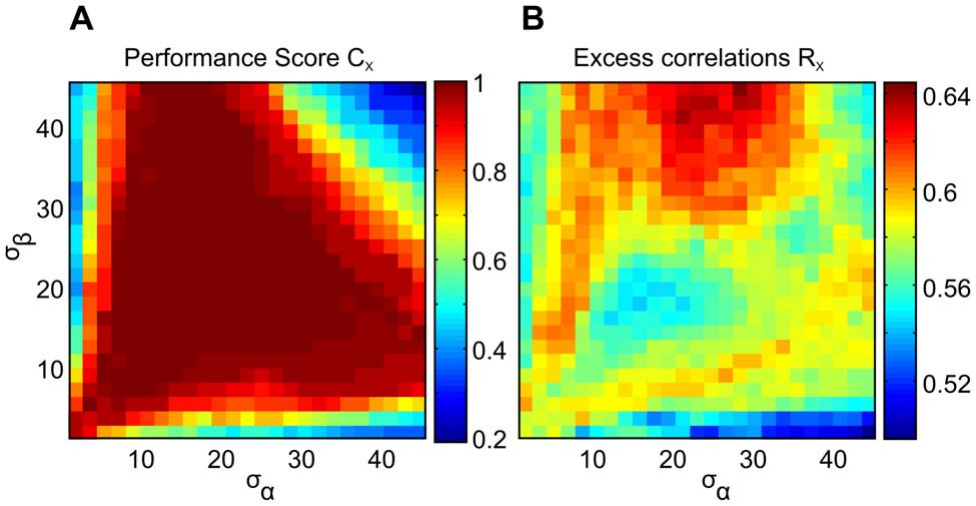
Comparing different measures for fitting the model to psychophysical data. (A) Performance score 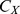 and (B) excess correlations 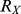 for different models 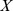 with independently varied association field parameters 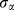 and 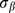.

The results shown in **Fig. 6** demonstrate that reproducing correlated human decisions is a stronger constraint for model evaluation than accomplishing performances. For this didactic example we held 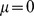 and 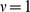 constant and only varied the association field parameters. While the performance score 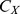 in **Fig. 6** A reaches one for a multitude of parameter combinations, correlation with human behavior 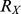 reveals a more distinct pattern where only few parameter combinations reach peak values. It can also be seen that varying these two parameters alone is not sufficient to reach the model benchmark of 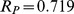. Although reproducing 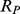 is a strong selection criterion, we note that surpassing mean performance at the same time is a necessary second criterion, because high values of 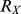 not always coincide with a sufficiently high mean performance (compare **Fig. 6** A with **Fig. 6** B).

**Figure 7:**
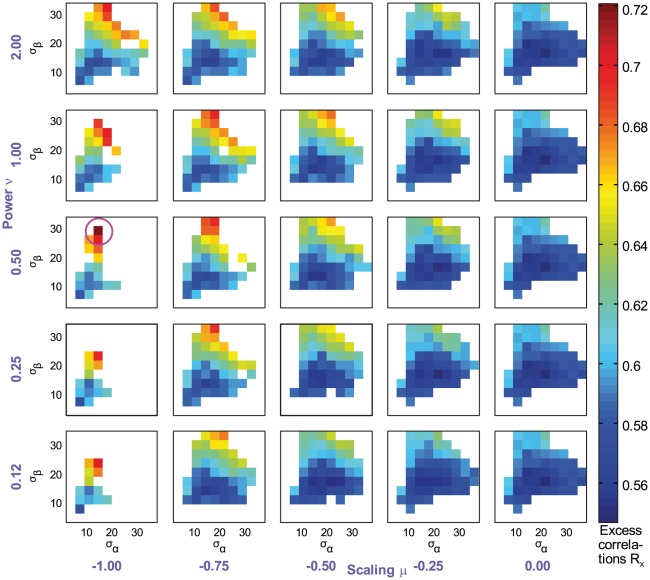
Searching for the best contour integration model 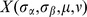 in a four-dimensional parameter space. Excess correlations 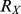 are shown in color code (see color bar on the right). Each subfigure encloses the results for one specific choice of the scaling parameter 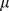 and power coefficient 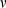, with 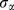 and 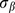 independently varied within the shown range. The parameter combination with highest 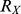 is enclosed with a purple circle, and combinations for which contour integration performance was inferior to humans (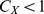) are left white.

We next varied the four parameters independently. Extensive simulations (the most relevant parts of the sampled parameter space are shown in **Fig. 7**) reveal one specific parameter combination, 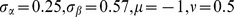, for which our best-performing model 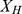 reaches 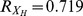, thus fully explaining human decisions for this experiment (sketch of AF and eccentricity scaling resulting from these parameters depicted in **Fig. 2** B,C, right graphs). Neither varying the parameters of the AF, nor varying the parameters of the eccentricity scaling alone yielded values of 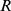 exceeding 0.63. From here on, we will refer to the model defined by this ‘best’ parameter set 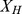 as the ‘optimal model’, in order to distinguish it from the original, ideal model.

This result is surprising, because a structurally simple model with only four parameters captures the full variety of human behavior in our experiment, which was tested with a multitude of different stimulus sets. The performance of this noise-free model is still superior to human performance (**Fig. 4** B, open circles), but much closer to the experimental data (**Fig. 4** A) than in the ideal observer (**Fig. 4** B, crosses).

In order to compare model behaviour to temporal aspects of contour integration observed in human subjects (**Fig. 5** A,B), one can link iteration depth in eqn. (3) to SOA in the experiment. For this purpose, we assume that linking contour elements in the brain bases on neuronal signals that propagate with a constant velocity from edge detector to edge detector (possibly over several relay stations or interneurons). One iteration (i.e. matrix multiplication) in the Bayesian algorithm eqn. (3) would then correspond to the time 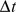 a neuronal signal needs to bridge the average distance 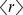 of two edge elements linked by this iteration. As total contour length is constant in the experiment, the average element distance is proportional to the reciprocal of the total number of contour edges 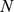, 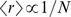, and performing 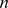 iterations then corresponds to real time 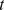 via 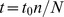. Heuristically, using smaller SOAs in the experiment is similar to a reduction in the number of matrix multiplications, which in turn is formally equivalent to computing the likelihoods for edges belonging to contours with less elements.

By choosing 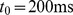, we assume that the largest SOA in the experiment corresponds to 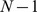 iterations (**Fig. 5** C,D). The temporal dynamics of the optimal model turns out to be remarkably similar to the time courses of human subject's performances for different SOAs (**Fig. 5** A,B). This indicates that the dynamics of human contour integration is at least compatible with a recurrent computation scheme.

### Predictions of the model

Model parameters and the dynamics of the recurrent algorithm make specific predictions for neurophysiological and behavioral variables.

**Figure 8:**
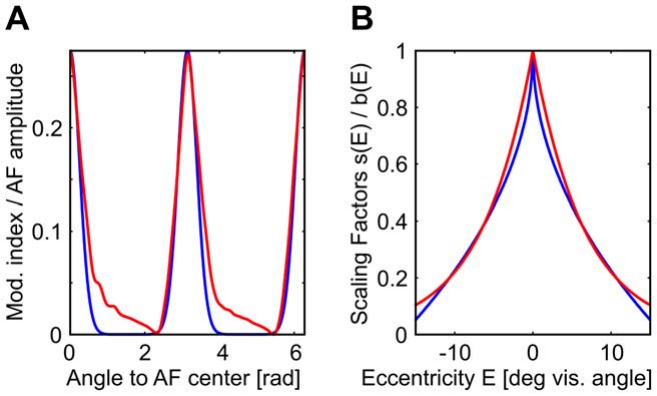
Comparing the model to data from independent experiments. (A) Comparison of association field parameters to electrophysiological data. Red, modulation index of firing rate of a cortical neuron to a preferred stimulus in dependence on the angular position of a second, flanking stimulus of same orientation (cross-section extracted from Fig. 2C in [23]). Blue, cross-section through optimal association field, scaled to the maxima of the red curve. (B) Comparison of attenuation of edge likelihood with eccentricity with psychophysical data. Red, sensitivity modulation 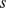 required to explain psychophysical edge detection thresholds in dependence on eccentricity 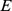, (from [24]). Blue, optimal likelihood modulation 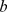. Parameters and equations see main text.

The parameters 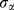 and 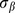 suggest a specific shape for the AF that fits the behavioural data best. The investigations of Kapadia et al. [23] provide independent data for a comparison of this shape to electrophysiological findings. They measured the modulation of the response (firing rate) of a cortical neuron in area V1 to an edge element within its classical receptive field in dependence on the presence of a second, flanking edge element presented at varying locations outside this region. The strength of this modulation reveals contextual interactions that could implement such an AF. Interestingly, the shape of this modulation curve at a distance of 0.5 degrees of visual angle from the receptive field's centre matches nicely with the shape of a cross-section through our AF with the optimal parameters (**Fig. 8** A).

A second comparison can be made with AFs extracted from labeled contours in natural images [16]. In order to approximately match the angular characteristics (**Fig. 3** B,C in [16]) and spatial extension (**Fig. 3** E in [16]) of the edge co–occurrence statistics, 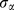 and 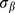 have to be reduced by a factor of about 2–2.5. The reason for this deviation might be rooted in the mean edge distance considered in [16], which is by about the same factor smaller than the mean distance used in our experiments. In fact, the largest distance considered in the edge co–occurrence statistics (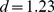 degrees of visual angle) is even smaller than the smallest edge distance in our experiments (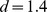 degrees of visual angle). Assuming that contour curvature is a critical parameter for contour integration, the maximum direction 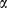 for which two edge elements are still integrated into a contour will depend on edge distance. In particular, if the mean distance between edge elements is reduced by a factor 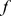, the maximum direction 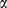 would then have to be reduced by about the same factor.

Parameters 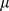 and 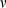 suggest a specific shape for the visibility of an edge, or the reliability of its neural representation, in dependence on visual field eccentricity. Also here, a comparison with independent data is possible: Foley et al. [24] quantified psychophysical thresholds for detecting edge elements at varying eccentricities in the visual field. This study computed a sensitivity modulation factor 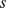 for a Gabor-shaped receptive field required to explain psychophysical detection thresholds. The dependence of this factor 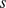 on eccentricity 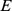 was well–described by 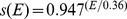. Again, comparison with the scaling function 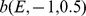 using the parameters of the best model 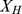, reveals a very close similiarity between the two curves (**Fig. 8** B).

Beyond comparing model properties to already existing experimental data, we obtained predictions which motivate further experiments. The probabilistic nature of our model requires stimulus evidence to be multiplicatively combined with recurrent feedback from neighboring edge elements. This feature is different from most current, biophysically motivated neural networks models performing contour integration by summing the corresponding inputs. Further simulations (not shown) suggest that a nonlinearity in synaptic integration of recurrent and feedforward inputs is indeed required for explaining human behavior [26], [27].

**Figure 9:**
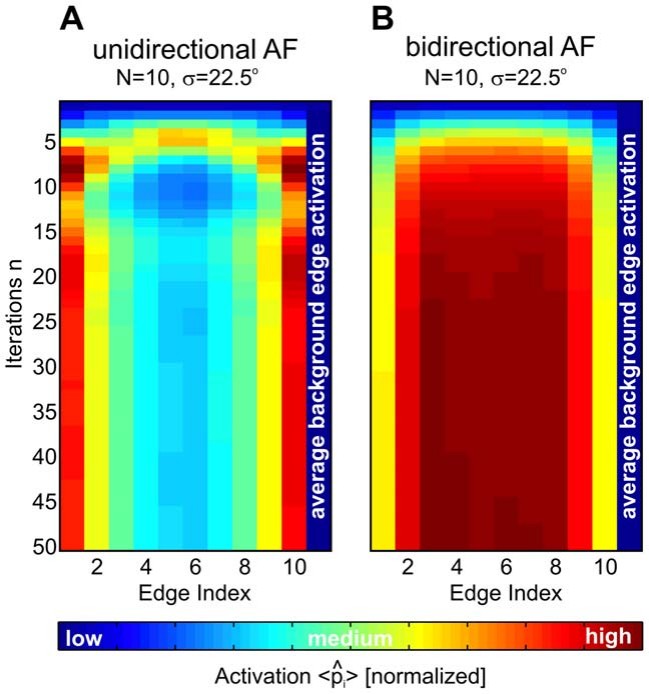
Predictions for different association field symmetries. Average likelihoods 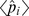 to be the starting element of a contour of length 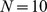, shown for all edge elements belonging to 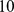
–element contours (left columns), and for background edges (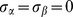). Vertical axis denotes iterations in eqn. (1), and the color scale is normalized to minimum/maximum likelihoods in each graph. (A) shows the corresponding dynamics for the optimal model which uses uni–directional AFs. For obtaining (B), we symmetrized the AF of the optimal model such that it became invariant to the directions of arbitrary edge pairs. For this bi–directional AF, simulations on the same stimuli as used for (A) were performed. If neuronal populations would encode these likelihoods 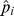, uni–directional interactions would cause highest activities at the end of a contour, while bi–directional interactions predict highest activities at center elements of a contour.

A further prediction derives from the unidirectional nature of contour creation, which suggests a similar unidirectional process also for contour integration. Unidirectionality significantly enhances performance in comparison to bidirectional interactions. In such a scenario, activation of neuronal feature detectors would spread into one direction along the contour, in contrast to classical contour integration models where association fields and functional interactions are not directionally biased. If neuronal populations would encode likelihoods for oriented image patches to be part of a contour, according to eqn. (3) these different coupling symmetries would predict different activation patterns for the populations receiving feedforward input from image patches which are parts of contours (**Fig. 9**). Specifically, unidirectional interactions would cause highest activity in neurons representing the end elements of a contour (**Fig. 9** A). In contrast, bidirectional interactions would cause highest activity at central elements of a contour (**Fig. 9** B). In addition, the model dynamics predicts oscillatory patterns which dampen over time until a stationary state is finally reached.

## Discussion

In summary, we proposed a model for contour integration whose parameters were calibrated to explain human decisions beyond average performances. In our experimental setting, it fully reproduces average human behavior. At the same time, the model possesses a well-defined probabilistic objective, i.e. it computes the likelihoods that observed edge elements belong to contours. For understanding recurrent computation in the visual system, our particular approach thus establishes a novel framework. Its distinctive feature is to quantitatively unite modeling and experimental data with a normative theory. If successful, such a framework allows to explictly specify a mathematically precise objective for a visual or cognitive function.

Qualitatively, the structure and – to a certain extent – the dynamics of our model are similar to models proposed by other studies [28], [29]: elementary feature detectors are linked by connections which are positive (i.e., enhancing activation), if the features are aligned colinearily in retinal space, and activation of feature detectors is propagated in parallel to other detectors over these links. We also confirmed that the shape of interactions emerging from our parameter search is indeed very close to physiological data [23]. A complementary idea was explored by Geisler et.al. [16]: instead of finding the ‘right’ shape of the AF by fitting a model to empirical data, they derived the corresponding statistics from natural images by computing the edge co–occurrence likelihoods from contours traced by human observers. As explained in the Results section, our AF has about the same properties as the edge co–occurrence statistics if it is properly rescaled for smaller edge distances. Geisler et al. also used the AF in a proabilistic model to predict human performance in a contour integration paradigm. In general, these predictions were qualitatively very good, but human performance was not always fully reached by their model. In an interesting extension of this work, Geisler and Perry asked human observers whether two edges at the border of an occluder belong to the same or different physical contours [15]. In this task, the subjects achieved a performance similar to an ideal observer constructed from the statistics of labeled contours in natural images.

One major advantage of our specific framework is that it extends beyond matching performance only. **Fig. 6** A clearly exemplifies that many different models 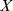 can meet this benchmark. In consequence, one particular model reproducing performance might not tell us very much about the real structures, parameters, and dynamics that underlie contour integration in the brain. By requiring a model to reproduce systematic deviations from this average behaviour for individual stimuli, we exploit an additional source of information (beyond average task difficulty) which helps to narrow down the plethora of models considerably (**Fig. 6** B). To pinpoint the essence of this idea: by demanding models to deviate from ideal behaviour, and to make the same systematic errors as humans, we make them explain the data better. In the specific setting used in this work, systematic errors are explained by both, the decrease in edge visibility with eccentricity, and the particular parameters (shape) of the AF. Neither one of these two factors alone can explain the excess correlations between human observers to their full extent. In some examples where observers made errors, the target contour was located in the periphery, while a smaller ‘chance’ contour near the fovea was apparently more salient. In other cases where consistent errors among observers showed up, it was difficult to unambiguously determine the particular stimulus feature that led to the erroneous decisions. A more thorough analysis will require to determine where the observers ‘look’ if they search for a contour.

A second advantage of our approach is that during the search for a ‘better’ model, we remain within a class of probabilistic models with a well–defined objective: computing the probability of edge elements to belong to contours, whose statistical properties are quantified by the model's parameters 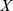. By reproducing behaviour to the maximally possible extent, we finally arrive at a model which is no longer optimal with respect to the arbitrarily chosen task, but optimal with respect to a similar task and under the given constraints. In a broader context, our observations fit well to similar probabilistic frameworks that explain visual illusions, i.e. apparent failures of our visual system, by the idea that the current task does not match the objective or priors of visual information processing [13].

In addition to this major conceptual point, our work sheds further light on the nature of contour integration: First, the dynamics of the iterative integration process in the model looks very similar to the performance of human observers in dependence on SOA time (**Fig. 5** A,C and **Fig. 5** B,D). A similar dynamics which also saturates after only a few recurrent cycles has been observed in a biophysically realisitic model with long–range excitatory interactions [28]. Somewhat counterintuitively, our model also reproduces the experimental fact that longer contours are perceived earlier, if decisions are based on the outputs of each iteration. These results underline that our framework is compatible with iterative information integration in the visual system. Second, we found that human observers have a performance considerably higher than chance level even if the contour was presented for only 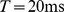. This result indicates that contour integration is a fast perceptive process [30], which further constraints putative neural mechanisms.

We expect that our model generalizes well beyond explaining only our experimental data and reproducing specific observations [23], [24] because the almost perfect match between model and human behavior does not result from overfitting: We used only four independent parameters to explain decisions for more than 2000 stimuli from 42 different contour ensembles. Moreover, we obtain more information from the fit than we initially put into the model: The ideal contour observer (which is the ‘inversion’ of the respective contour–generating process for each contour ensemble) is actually the worst in explaining the human data. Only after including realistic constraints as e.g. restricting the integrator to one association field, we were able to reproduce our psychophysical data. It will thus be interesting to see how our optimal model will perform on different contour integration problems. For example, comparison with the Geisler et al. data [16] suggests that for smaller mean edge distances (i.e., denser Gabor fields) than in our experiment, the angular parameters of the AF have to be rescaled. Furthermore, the model currently does not capture effects where cues in features other than the relative alignment of edges modulate contour integration. Such features include colour [31], [32], contrast [33], or spatial frequency [7], [34]. It is not clear whether varying other features of the background or of the distracter elements will impair contour integration [7], or have only a negligible effect on performance [35]. A natural extension of our model would use an extended parametrization (i.e. orientation, spatial frequency, and colour instead of orientation only), and introduce interactions between similar feature combinations, thus mimicking the physiological observation that neurons with similar response properties have a higher probability to be connected.

A unique feature of the model is the directionality of its interactions, which is inherited from the directedness of the contour generation process [13]. For understanding its implications, consider for example contour integration along a straight, horizontal sequence of aligned horizontal edge elements: In a ‘classical’ contour integration model, each edge activates one feature detector with preferred horizontal orientation. Activation from this detector then symmetrically spreads to the left and to the right to the neighbouring detectors (bidirectional interactions). In contrast, in the probabilistic model each edge activates *two* detectors with the same preferred horizontal orientation. One of these detectors will then spread activation only to the left neighbouring detectors, while the other detector will spread activation only to the right neighbouring detectors. There is no crosstalk between the two detectors. Hence contour integration is performed by two independent processes propagating in parallel into two opposing directions along the contour (unidirectional interactions).

From a computational point of view, such unidirectional interactions are more efficient by avoiding false positives in contour detection [36]. For example, they effectively suppress ‘contour’ configurations with changes in direction by 180 degrees, such as two circle segments that are attached tangentially at one of their ending points. In fact, comparisons of further simulations (not shown) with our psychophysical data suggest that bidirectional couplings normally used in contour integration models can not even explain human contour integration *performance* [26], [27].

Is our contour integration model biophysically plausible? Its interactions needed to perform contour integration could be mediated by orientation–specific connections between cortical neurons. Examples for such connections, which preferentially link neurons with similar orientation selectivity, are horizontal long–ranging axons within primary visual cortex (V1) [37]–[39], or backprojections from secondary visual cortex (V2) to V1 [39], [40]. Our results show that one single, ‘general purpose’ association field is sufficient to quantitatively explain human behavior in response to stimuli generated from multiple AFs. Thus in principle, only one ‘set’ of cortical long–ranging axons with a geometry matching the AF of our optimal model is sufficient to perform contour integration in the brain, if this structure is used iteratively in a recurrent computation. However, the variety of geometries and length scales associated with these connections in different animals makes it currently difficult to determine the real extent to which they support contour integration. In addition, implementing unidirectional interactions anatomically would require two distinct neural populations with similar preferred orientations, but asymmetric dendritic trees. Such a structure currently seems to be in conflict with experimental evidence (homogeneous populations, largely symmetric dendritic trees, as e.g. shown in [37]), although its existence can not be fully excluded from these studies.

Regarding the dynamics of contour integration, the probabilistic model performs inference by iteratively using parallel computations that can easily be emulated by neural networks. For example, the matrix–vector multiplication 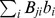 can be re–interpreted as the summation of pre–synaptic afferents 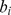, weighted by the synaptic efficacies 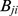, on the dendrites of a post–synaptic neuron 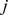. These interactions lead to a modulation of an edge detectors' activity by the presence of other edges in its neighborhood. It is known that starting from V1, neural responses indeed become modulated by the context of a stimulus within their ‘classical’ receptive field [41], [42]. This modulation can enhance firing rates for colinear edge configurations [43]–[45], and can cause neurons to be active also in response to illusory contours [46]. One problem with the accumulated evidence for contextual modulations as putative signatures for contour integration processes in cortex is their controversity. For instance, substantial suppressive effects for colinear edge arrangements [47], [48] have also been observed. In addition, firing rate modulations are often weak or critically depend on the exact stimulus configuration, which stands in contrast to the strong and robust effects established by psychophysical studies. Despite this sometimes confusing empirical evidence, **Fig. 8** A demonstrates that modulation of activity induced by neighboring contour elements in the optimal model matches very well to electrophysiological data. In addition, neural dynamics might also provide a more realistic mechanism for establishing unidirectional contour integration than requiring a directed anatomical substrate as discussed above. Unidirectionality can be realized by volleys of activity, which propagate along the neural populations activated by the contour's edges [49]. Refractoriness of neurons would be the basic mechanism that ensures that activation waves can not easily reverse direction. One possibility to test our prediction of an unidirectional process underlying contour integration is to perform massively parallel recordings in animals performing contour integration. Focusing on the activation dynamics of neurons whose receptive fields cover distinct elements of the contour would allow to directly observe activity waves propagating in a certain direction. An alternative, but more indirect test could focus on the specific predictions (**Fig. 9**) made by models with different coupling symmetries. Here it would be sufficient to record and compare neural activity of neuronal populations representing central edges and starting/ending edges of contours, respectively. Experimentally, this scenario is technically less demanding as it only requires single–unit recordings. Unidirectionality then predicts highest activity at starting/ending edges, while bidirectional models predict the opposite behaviour. Although there is yet no experiment addressing this issue, recent neurophysiological recordings of V1 neurons [50] which were stimulated by edge elements being part of a contour show a very similar time course of activation: a strong transient response, followed by a dampened oscillation that relaxates into a sustained activation level.

A remarkable difference to more ‘standard’ neural networks [28], [29] is that the afferent input (i.e., evidence from the stimulus) and the recurrent feedback (i.e., linking probabilities between edges) are combined multiplicatively instead of additively to produce a unit's output. The utility of such a non–linear operation for contour integration was indeed suggested by previous modeling work on feature integration [51]. It is known that non–linear computations on synaptic inputs are performed as early as from LGN and primary visual cortex on [52]–[54], and it is possible that these non–linearities provide the substrate required to compute the AND-like operations necessary for implementing Bayesian inference.

Evolution has adapted information processing in the brain to serve many objectives still awaiting discovery. While for some simple and very fundamental tasks, experiments could demonstrate that perception can be described as optimal inference [11], [12], there are many reports from psychophysics that suggest the visual system to not operate optimally. The notion of optimality, however, is (a) relative to some external criteria (i.e., the task design) that must not neccessarily be evolutionary relevant, and (b) need to take constraints into account. These considerations might have prohibited the application of normative approaches to more complex visual functions, as e.g. the perception of objects. In our work we overcame these difficulties by starting with a probabilistic framework whose basic mathematical structures were motivated by known properties of human contour integration. This framework provides both, a task design for experiments or simulations, and an initial suggestion for a computational model. Introducing realistic constraints and fitting the model's structure to human decisions finally revealed that also human contour integration can be well described as optimal inference on a sensory stimulus. Moreover, our results demonstrate that such an integrative approach may generate fundamental predictions about neural mechanisms that are difficult to obtain in a pure bottom–up modelling approach.

## Methods

### Generative model for contours

We adapted the framework by Williams and Thornber [13] to contours of finite length which are generated by a Markov process: Let 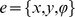 denote an edge element with associated direction 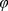 at coordinates (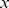, 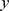), in two-dimensional space. If a contour passes through edge 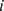, 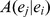 defines the probability that the contour will pass next through edge 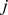 (transition probability or ‘association field’). Contours of length 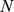 are generated by first positioning a starting edge at a random position, and then sampling a sequence of 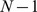 further edges from the association field 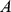.

### Defining an association field

For a meaningful definition of contours, 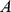 should possess a translational symmetry (same probability for creating a specific edge configuration at different locations), a rotational symmetry (same probability for creating an identical, but rotated contour), and a reversal symmetry (same probability for creating a contour with the reverse sequence of edges) [13]. These symmetries effectively reduce the six-dimensional conditional probability distribution 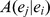 to a three-dimensional function 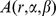 which depends on the parameters 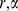 and 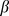. For two edges 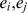, these parameters are given by the coordinate transformation
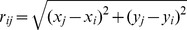
(6)

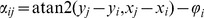
(7)

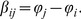
(8)

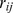
 is the Euclidean distance between edges 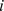 and 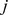, 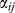 the angle under which an observer at edge 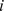 looking into the direction 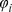 views edge 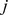, and 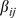 is the difference between the directions of edges 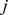 and 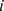 (see **Fig. 2** A).

We defined 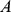 as a product of a radial part 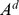 and an angular part 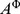 via 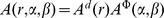. The radial part will be described in the next subsection. The angular part was parametrized as a product of von–Mises functions 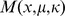 that correspond to Gaussian distributions defined on a circular support,
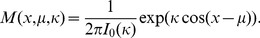
(9)

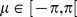
 is the circular mean, 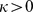 a concentration parameter (length scale), and 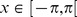 the angular variable. 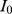 is the Bessel function of the first kind, of order 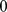. By the transformation 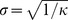, 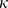 is related to the width 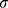 of a Gaussian distribution. The parametrization of 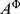 then reads
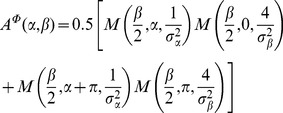
(10)

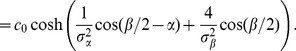
(11)We used 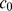 to abbreviate a normalisation factor given by
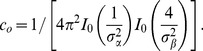
(12)This particular choice of 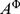 (**Fig. 2** B, centre) implements two important principles for association fields, namely (I) link probability decreases on a length scale 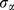 with the distance from a co-circular edge configuration with 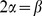 [19], and (II) link probability decreases on a length scale 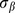 with increasing curvature 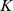 (for co-circular edge configurations with inter-edge distance 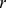, 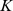 is directly related to 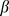 via 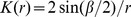).

### Hiding contours among distracters

The idea of this contour integration experiment is to hide a contour among randomly oriented distractor elements, and to force a human observer to use the relative alignment between the edges as the only cue to find the contour. This implies removing all other hints about the location of the contour, as e.g. element distances or densities, from a stimulus. For this purpose we employed an improved procedure similar to the algorithm proposed by Braun [36]: Starting from a regular positioning of edge elements filling the background around a contour, these elements are subjected to a Brownian motion until a dynamical steady state is reached. Typically this procedure yields an edge distance distribution 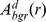 between background elements which differs from the contour edge distance distribution given by 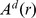. We therefore replaced our initial 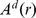 by 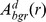, and repeated the whole procedure iteratively until the (I) background-background edge distance distribution, (II) background-contour edge distance distribution, and (III) contour-contour edge distance distribution were identical.

When generating a contour with large curvature in the first place, it could happen that two distant edges will overlap when they are rendered for stimulus display as finite-width Gabors. We omitted this problem by randomly permuting the sequence of relative angles 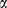, 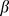 and distances 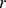 between subsequent contour elements until any overlap vanished. By this policy we prevent giving unwanted cues to the location of a contour, while at the same time conserving the pairwise edge statistics of contour ensembles implied by a specific association field.

### Detecting contours by inference

In our paradigm, a contour is placed either in the left or in the right hemifield of a stimulus, and hidden among distracters. An observer has then to decide on which hemifield the contour has been placed (two-alternative forced-choice). We now derive an optimal contour observer for this situation:

A stimulus 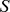 decomposes into a part 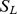 on the left, and a part 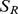 on the right hemifield. Each part 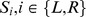 consists of a set of edge elements, in which any combination of 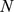 edges could correspond to the hidden contour. We call a specific edge combination a *contour configuration* 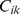, which is an ordered set of 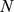 edge elements. Index 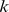 runs from 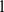 to 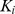, which is the total number of all different, putative contour configurations in stimulus part 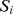. Note that different configurations 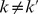 may be composed of the same edge elements, but in a different ordering.

We now compute the probability that a contour placed into 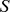 is contained in stimulus part 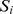. To simplify notation, we denote the contour configuration we are looking for with 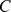, and the (unordered) set of background elements with 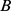. We have to sum over all 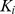 possible contour configurations:
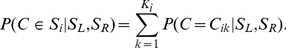
(13)The right hand side can be expressed in terms of the likelihood 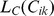 that configuration 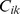 was obtained from the generative contour model,
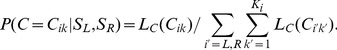
(14)Next we express the likelihood 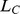 in terms of the association field 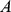. With 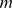 and 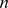 denoting two arbitrary edges in stimulus part 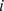, we define the components 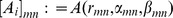 of likelihood matrices 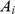 by sampling from the association field 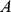. For a specific configuration 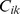 where the index sequence 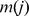, 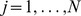 defines the succession of edges, 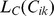 can be written as
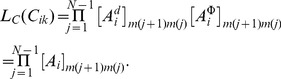
(15)In the final step, we split the sum over all edge configurations 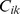 into a sum over all edges 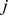 where a contour can start, and a sum over all edge configurations that have the same starting edge 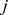,
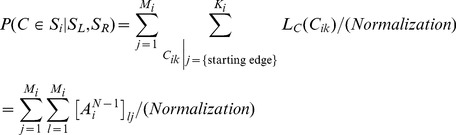
(16)with the appropriate normalization terms from the denominator in eqn. (14). Here we introduced 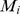 to denote the total number of edge elements in hemifield 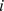. If the probability for a contour in a specific hemifield is 1/2, the ideal contour integrator will estimate that the contour was placed on the left hemifield if 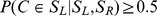. Thus eqn. (16) corresponds to eqn. (2).

### Stimulus sets

For the psychophysical experiments and model simulations, we used 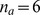 different parameter sets 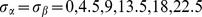 degrees for the shape of the association field eqn. (11). We used 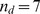 different numbers of contour elements (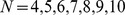) while holding the length of the contour approximately constant, which causes the average inter-element distance 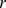 to be proportional to 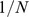. All combinations of these parameters gave a total of 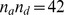 stimulus conditions.

For each stimulus condition, 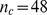 contours (targets) were generated and each embedded into randomly oriented background elements according to the procedure outlined above (with 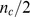 contours on the left hemifield and 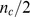 contours on the right hemifield). The inter-edge distance statistics approximately followed an exponentially decaying function. While our procedure suppresses all first-order cues from the inter-edge distance statistics, there is a remote possibility that observers might use second- or higher order cues to locate the contour. This problem was avoided by generating a second contour path on the hemifield opposite to the target contour, but randomly choosen orientations for the edge elements. For each of these stimuli, masks were generated with edges of random orientations at the same positions.

For stimulus presentation, contour and mask stimuli were rendered by placing Gabor elements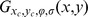 with spatial extent 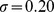 degrees visual angle (8 pixels), wavelength 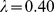 degrees visual angle(16 pixels), random phase 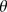, and orientation 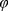 centered at the corresponding positions 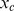 and 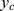. 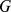 was defined as
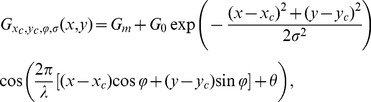
(17)with 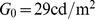 denoting the contrast and 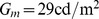 the mean background luminance. The mean distance between the contour elements for 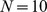 corresponds to 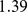 degrees visual angle.

### Psychophysical experiments


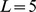
 subjects (2 female, mean age 29.4 years) participated in the two-alternative forced choice (2-AFC) experiment. All had normal or corrected-to-normal vision. They sat 80 cm in front of a gamma-corrected, 21–inches CRT screen (1152×864 pixels, 100 Hz refresh rate). Each trial started with the appearance of a small fixation spot in the display center.

After a fixation period of 1 s, a contour stimulus was presented which was followed by its corresponding mask after a time  (stimulus onset asynchronies, 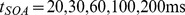). Presentation of the mask lasted for 500 ms, followed by a blank screen. Observers were instructed to indicate the hemifield where the contour had been displayed (left or right) by pressing one of two response buttons during the blank period at the end of each trial. Responses occuring too early or too late (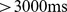) after mask offset were rejected. In summary, each observer had to detect 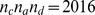 contours for each of the five SOAs. For assessing decision correlations between subjects, we used the same 2016 stimuli for different observers, but presented them in a randomly interleaved order which was different for each subject.

### Statistical methods

We evaluate the similarity between a model 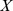 and our 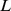 human observers by comparing their mean contour detection performances and their individual decisions.

Let 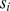 denote the score of an observer 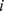 for one stimulus, with 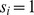 if the hemifield with the contour was identified correctly, and 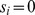 otherwise. With 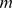 indexing one out of 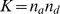 stimulus conditions, the total number 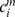 of correctly detected contours is given by 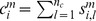. The percentage 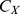 of conditions in which model 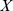 has an equal or higher contour detection performance is then given by
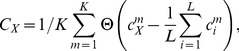
(18)with 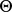 denoting the Heaviside function. 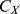 is our first benchmark for comparing models to humans.

Next we consider the number 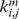 of identical responses of two observers 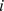 and 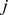 (which could either be two humans, or one human and one model 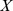), in stimulus condition 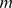,
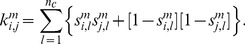
(19)We will now compare this value to the probability 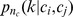 to obtain 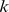 identical responses, provided that in total, 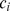 and 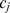 contours were detected correctly by observer 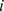 and 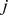, respectively. The basic assumption hereby is that contour detection errors are made independently of a specific stimulus within a stimulus condition 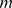. 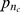 is easily computed by considering the number of possibilities how 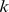 identical responses can be distributed among the 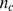 stimuli, while holding 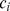 and 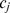 constant. Introducing 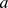, which is related to the other variables via 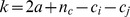, we obtain
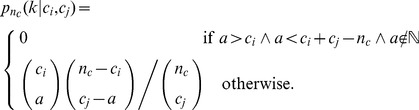
(20)


We finally compare the expected distribution of identical responses 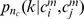 with the actually measured value 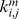 by computing the total probability 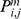 to obtain a value 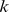 which is equal or lower than 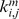,
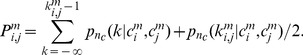
(21)Because 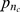 is a discrete probability distribution, we need to add a continuity correction for 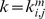 (last term). This term ensures that 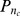 is on average 0.5 when 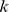 is drawn from 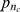. The average of 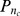 over all possible observer combinations 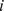 and 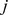, and over all stimulus conditions 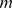, yields a number 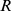 which is larger than 0.5 if observers' decisions are more strongly correlated than can be expected from our independency assumption. For the human observers in our experiment we obtain 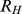 according to
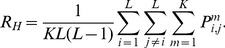
(22)The decisions of a specific model 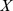 are compared to all human observers via
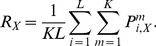
(23)For judging the similarity between a model 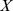 and human observers, we can not directly compare 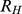 and 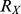: as argued in the main text, humans are subject to decision noise, but the model 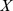 is not. Therefore the model will give identical responses in all repetitions of the same trial, while two humans would give different responses even if they had the same objective. Hence if we find a model 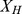 which perfectly explains human behavior, 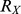 will always be larger than 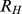. To remove this statistical bias, we construct 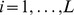 hypothetical, noisefree human ‘prototypes’ 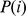 from the majority vote of the 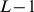 human observers 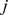 with 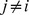. The decisions of these prototypes are thus given by
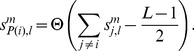
(24)By comparing the prototypes 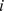 to their real human counterparts 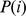, using the statistical methods as described above, we obtain
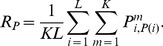
(25)

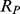
 defines our second benchmark for comparing models to humans: If 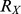 approximates 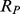, the corresponding model reproduces both, the nature and the amount of correlations in human behavior.
